# Supplementary material for: Understanding the adsorption process in ZIF-8 using high pressure crystallography and computational modelling
Source: Nat Commun. 2018 Apr 12;9:1429. doi: 10.1038/s41467-018-03878-6 (PMC5897325; doi:10.1038/s41467-018-03878-6)
Supplement: Supplementary file 1 — Supplementary Information [file 41467_2018_3878_MOESM1_ESM.pdf]

Supplementary Information

Understanding the adsorption process in ZIF-8 using high pressure crystallography and computational modelling

Claire L. Hobday *et al.*

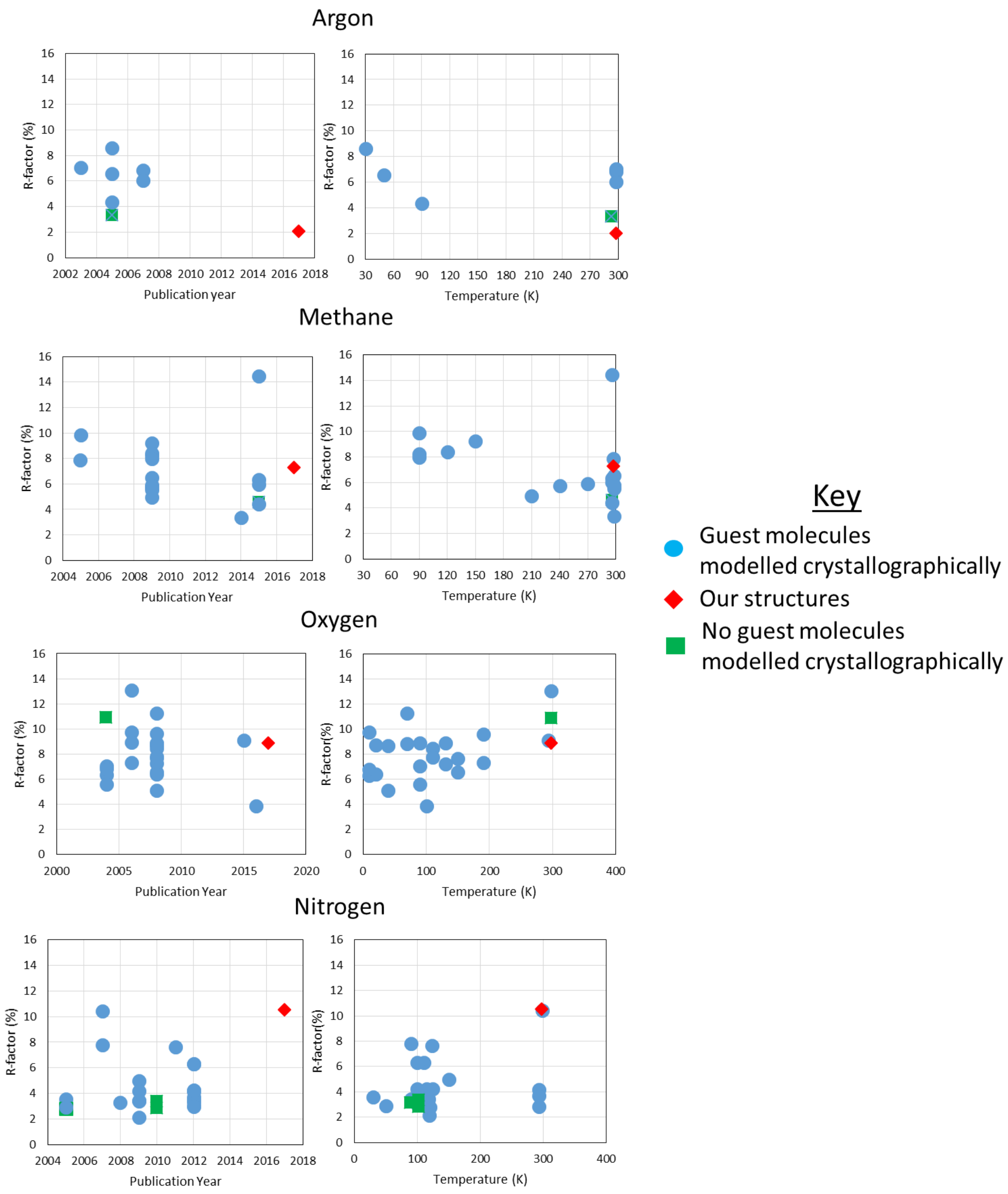

Supplementary Figure 1: Plots of gas included MOF structures in the CSD for the gases Ar, CH_4, O_2 and N_2. Using the new MOF subset of the CSD – a database which contains 79526 MOF structures (Feb 2017) - searches of gas containing MOFs could be efficiently achieved.[1] Of the gases studied in this paper, a CSD search highlighted that there were 7, 19, 27 and 24 single crystal structures of MOFs containing Ar, CH_4, N_2 and O_2 – a total of 77 structures. Of these 77 structures, only 6, 18, 24 and 23 actually contain refined molecules within the pores, a total of 71 datasets (0.08 % of the MOF subset). The other structures use the Platon SQUEEZE algorithm to estimate the gas content within the crystal.[2] Of the remaining structures, only 17 structures were collected at room temperature–3, 9, 3 and 2 for Ar, CH4, N2 and O2, respectively.

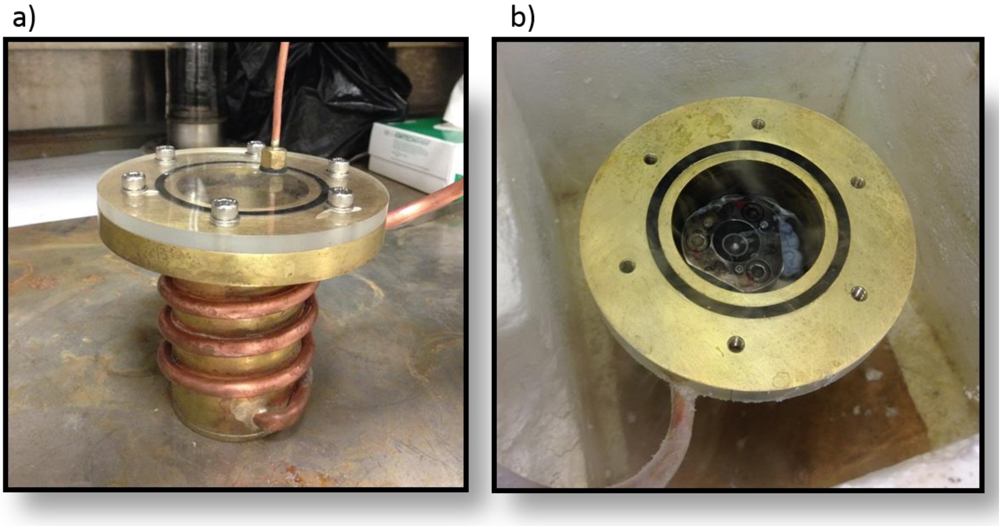

Supplementary Figure 2: Cryogenic loading chamber set up: a) side view showing the brass coils through which the gas is pumped into the chamber, b) top view showing the inside of the chamber with a DAC immersed in liquid CH_4, with the whole set up surrounded in liquid nitrogen in a polystyrene box.

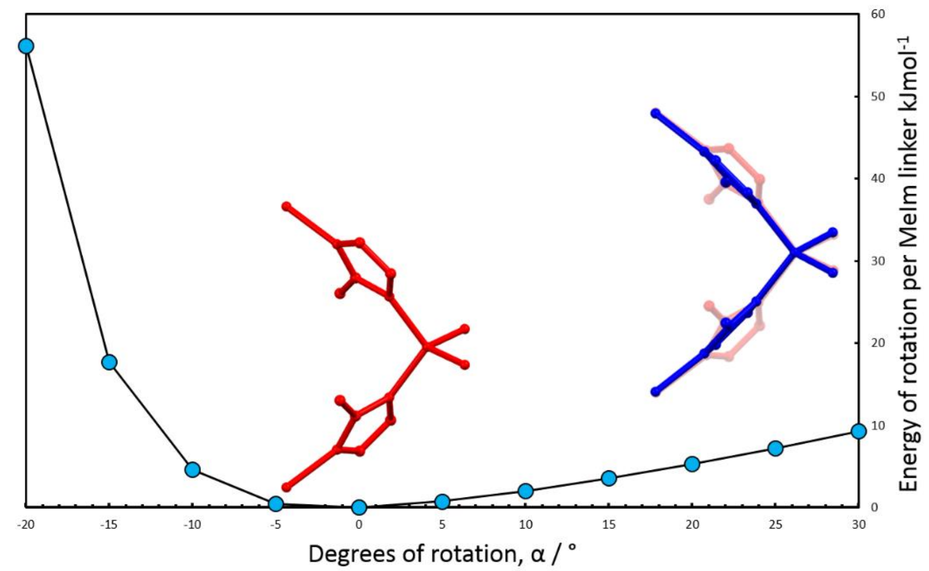

Supplementary Figure 3: ZIF-8 potential energy upon rotation of MeIm linker from ZIF-8AP (red structure) to ZIF-8HP (blue structure) in 5 degree increments.

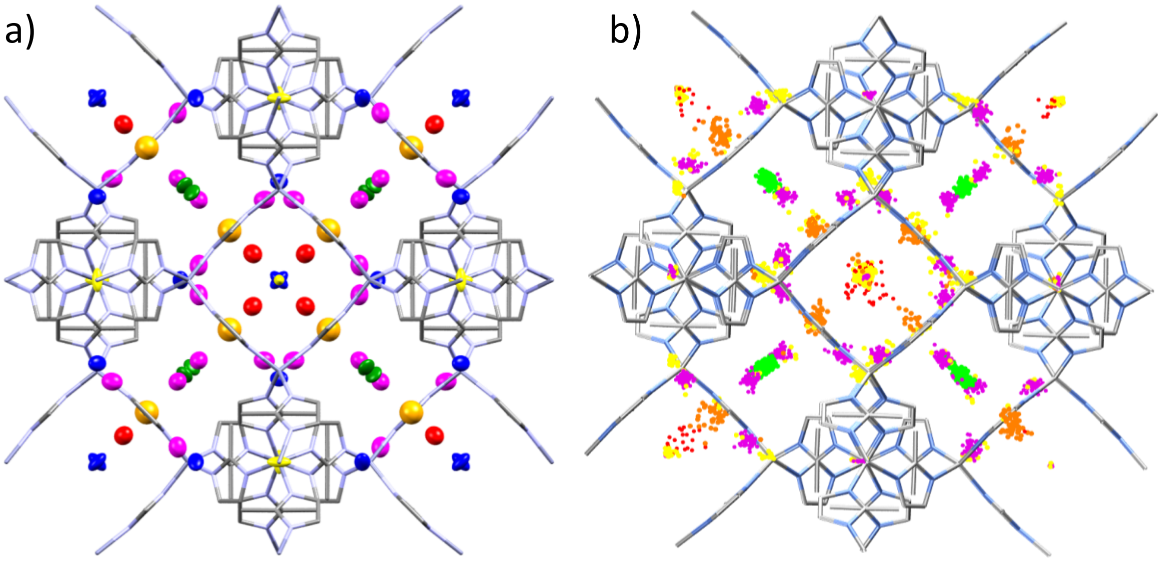

Supplementary Figure 4: **(a) Crystallographically determined CH_4 adsorption sites in ZIF-8 at 1.40 GPa as shown on a 6MR window for CH_4-1 (green), CH_4-2 (red), CH_4-3 (yellow), CH_4-4 (dark blue), CH_4-5 (orange), CH_4-6 (magenta). (b) CH_4 sites determined from GCMC simulations, where the colours correspond to energy levels that are colour coded in Supplementary Figure 5.**

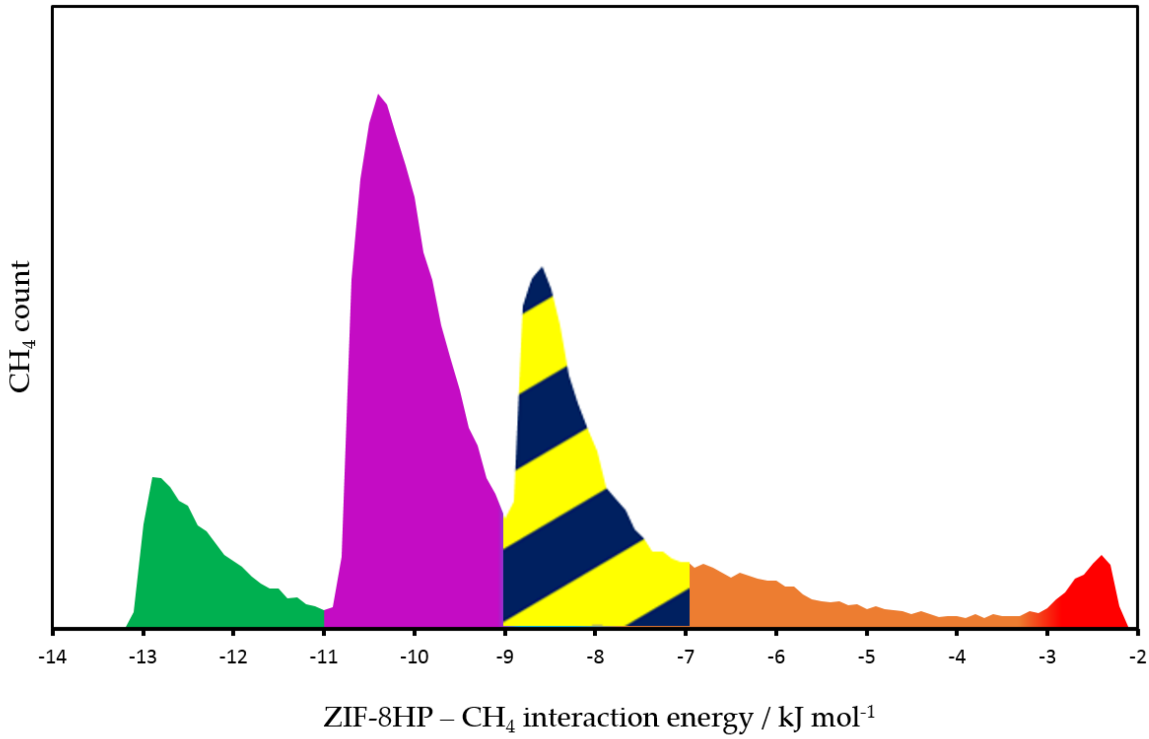

Supplementary Figure 5: **Histograms of guest- framework interaction energies at 1 bar (0.0001 GPa) of guest during GCMC simulation in ZIF-8-CH_4 using ZIF-8-HP. The coloured sections show the area of each peak in the plot and each colour is representative of the colour of each adsorption site in crystallographic sites. CH_4-3 and CH_4-4 cover the same energy range.**

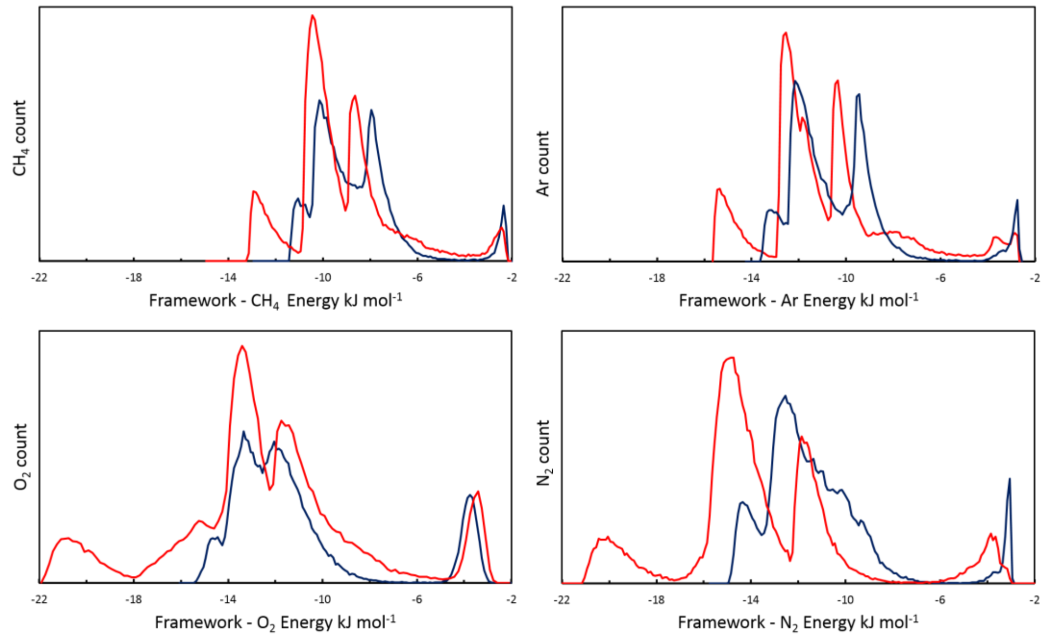

Supplementary Figure 6: Histograms of guest- framework interaction energies at 1 bar (0.0001 GPa) during GCMC simulation in ZIF-8 clockwise from top left ZIF-8-CH4, ZIF-8-Ar, ZIF-8-N$_2$, ZIF-8-O$_2$ Red plot shows interactions with ZIF-8-HP, blue plot shows interactions with ZIF-8-AP. Each peak normally corresponds to an adsorption site (although sometimes two different adsorption sites can have similar fluid-framework interaction energies). Analyses of the energy histograms of the two phases of the framework explain why the framework undergoes a transition: the interaction energies of each sorbate with ZIF-8 is lowered by ~ 3 kJ mol^{-1} (i.e. more negative and favourable) by switching to ZIF-8HP, and in the diatomic cases by up to 7 kJ mol^{-1}, so any penalty for the framework rotation (as calculated by DFT) to the HP phase must be outweighed by the favourable adsorption sites. Although the framework for ZIF-8 was once assumed to be rigid and the phase transition has yet to be seen upon variable temperature studies which could provide enough thermal energy to undergo linker rotation transitions, it has been shown here that the interaction with guest molecules induce linker rotation.

Supplementary Table 1: Pore content expressed per unit cell (uc), solvent accessible pore volume (SAV), and the different phases of ZIF-8 loaded with CH_4, O_2, N_2 and Ar as PTM on increasing pressure. The ZIF-8-HP phase is highlighted with an *.

Pressure (GPa)	CH_4 (mole/uc)	SAV (Å^3)	θ (°)
0.00	n/a	2497	65.1
0.30	26	2580	58.7
0.50	33	2551	58.9
0.70*	89	2657	86.6
1.10*	113	2710	87.4
1.40*	66[a]	2586	87.8

Pressure (GPa)	O_2 (mole/uc)	SAV (Å^3)	θ (°)
0.00	n/a	2514	65.1
0.21	26	2487	64.9
0.50	72	2457	66.7
0.75*	85	2522	87.2
1.20*	66[a]	2356	86.9
2.00*	33	2255	86.6

Pressure (GPa)	N_2 (mole/uc)	SAV (Å^3)	θ (°)
0.00	n/a	2497	65.1
0.21*	37	2548	86.2
0.74*	41	2525	88.0
1.03*	54	2512	87.7
1.33*	61	2402	82.8
1.85*	62	2364	86.2
2.72*	62	2223	89.3
3.25*	66[a]	2054	89.2

Pressure (GPa)	Ar (atoms/uc)	SAV (Å^3)	θ (°)
0.00	n/a	2497	65.1
0.75*	53	2458	86.7
1.20*	66[a]	2405	87.3
1.50*	51	2256	87.7

SAV = solvent accessible volume, calculated using PLATON.[2] Diameters of 4MR and 6MR calculated using the void analysis routine in Mercury, (grid spacing of 0.2 Å).[2] [a]Atoms or molecules per unit cell calculated from refined guest molecule content (otherwise estimated *via* PLATON SQUEEZE).

Supplementary Note 1: Effect of Pressure on the Framework Geometry and Pore Content of ZIF-8 using CH_4, O_2 and N_2 and as PTM

In a similar manner to CH_4, on loading ZIF-8 in O_2 at 0.21 GPa, θ also decreased (from 65.1° to 64.9°) (see **Supplementary Table 1**). This small rotation caused the 4MR and 6MR windows to increase and decrease from 0.8 Å to 0.90 Å and 3.0 Å to 2.9 Å, respectively. This was accompanied by an increase in pore content of ~26 O_2 molecules/uc. On increasing the pressure further to 0.50 GPa, θ increased to 66.7° and coincided with a further increase in pore content (~72 O_2 molecules/uc), the 4MR and 6MR diameters remained unchanged. On increasing pressure to 0.75 GPa, θ increased to 87.2° as a result of the transition to the ZIF-8-HP phase (with an associated framework rotation energy penalty of 6.8 kJ mol^{-1}), resulting in the largest uptake of O_2, (~85 molecules/uc). Both the 4MR and 6MR diameters increased to 2.2 Å and 3.6 Å, respectively, as a result of the transition. On increasing pressure to 2.0 GPa, the 6MR diameter decreased (along with the unit cell volume) as the ZIF-8HP phase was compressed. For ZIF-8 loaded in both CH_4 and O_2 PTM, it was possible to see the effect the guest had on the framework before transforming to ZIF-8HP, as for both the MeIm linkers rotate to lower θ values before transforming to ZIF-8-HP (resulting in an increase in θ). Although not explicitly mentioned in the original HP study of ZIF-8 using MeOH/EtOH as a PTM, there θ also decreased on going from ambient pressure to 0.18 GPa (from 64.3° to 59.2°).[3] On increasing pressure further from 0.18 to 0.96 GPa, θ increased to 59.8° before transforming to ZIF-8HP (where θ increased to 89.7°). This decrease in θ would therefore appear to be a general trend observed during the diffusion of fluid molecules into ZIF-8-AP prior to the transition to ZIF-8-HP.

In the case of N_2 and Ar loaded ZIF-8, the framework had already undergone the transition to ZIF-8HP upon loading (at 0.20 and 0.75 GPa respectively), resulting in an increase in θ from 65.1° in the ambient pressure structure to 86.2° and 86.7° respectively, with similar energy penalties for the framework rotation (of ~6.5 kJ mol^{-1} per linker). Both were accompanied by an increase in pore content (see **Supplementary Table** 1). The diameters of the 6MR windows increased from 3.0 Å to and 3.4 and 3.3 Å for N_2 and Ar respectively, while the 4MR windows diameters increased from 0.8 to 2.5 Å for both gases. On increasing the pressure further to 0.74 GPa with $N_{2,}$ the diameters of the 6MR windows increased to a maximum value of 3.5 Å, above this pressure the 6MR window diameter decreased in size along with the unit cell volume as the ZIF-8HP phase was compressed, whereas θ continually increased to a maximum of 89.2°

at 3.25 GPa (with an associated rotation penalty of ~7.5 kJ mol^{-1}, increasing by 1 kJ mol^{-1} from 0.20 GPa). The equivalent trend was seen in Ar loaded ZIF-8, where above 0.75 GPa the 6MR window diameters decreased along with the unit cell compression, with θ continually increasing to a maximum of 87.7° at 1.50 GPa. The associated energy penalty for the framework rotation is ~7 kJ mol^{-1}.

Supplementary Note 2: CH_4 loaded ZIF-8 model and simulation details

At a pressure of 1.40 GPa the CH_4 content reached a maximum and the adsorption sites could be refined with anisotropic displacement parameters (ADPs). CH_4-1, CH_4-2, and CH_4-5 show elongation along one direction (shown in green, yellow and orange respectively in **Supplementary Fig. 4**). The directions are perpendicular to the 6MR window for CH_4-1 and to the 4MR window for CH_4-2 and CH_4-5).

The density distribution analysis for ZIF-8-CH_4 was similar to ZIF-8-Ar, with each CH_4 site found in its refined crystallographic position (see **Supplementary Fig. 4).** One difference in the two systems was the decreased interaction energy between CH_4 and ZIF-8 compared to Ar and ZIF-8, where each site was found to be a few kJ mol^{-1} less favourable in ZIF-8-CH_4 than ZIF-8-Ar (see **Supplementary Fig. 5**). This is due to the size of Ar compared to CH_4, and is explicit in the larger LJ parameters. Ar is often thought of as an inert gas, however the dispersion forces are quite substantial.[5]

Supplementary Methods

For CH_4 and O_2 loaded ZIF-8, diffraction data were collected before the phase transition occurred. However, due to the low guest content and therefore its diffuse electron density contribution, only the framework could be refined in the ZIF-8-AP phase. As a consequence, the pore content was modelled using the SQUEEZE algorithm in PLATON for all pressure points in all PTMs. After the phase transition to ZIF-8HP, when the electron density in the pores was sufficient enough to model and the data quality was at its highest, structural models were obtained for the framework and guest gas molecules this occurred at 1.40, 0.75, 3.25 and 1.20 GPa for CH_4, O_2, N_2, and Ar.

The SQUEEZE algorithm was applied (probe radius 1.2 Å, grid spacing 0.2 Å) to calculate the electron density in the pores and give an estimate to the number of guest species in the pore as a function of pressure. The number of guest molecules was corrected for the residual electron density in the ambient pressure data set and is shown in **Supplementary Table 1**, along with the crystallographically modelled number of guest molecules per unit cell. As PLATON calculates residual electron density across the entire pore region, one would not expect the results from refined occupancies from an atomistic model to be exactly the same as the electron density calculated using SQUEEZE, though it is recognised as standard method for modelling guest molecules when electron density is too diffuse.[6-8] For each gas studied in Table S1, one can see that the general trend shows an increase in pore content, with the number of molecules per unit cell increasing with increasing pressure – a sensible result – however, the absolute value must be taken with some caution. The SQUEEZE data is contained in the SI to show completeness and transparency in the quality of the data.

Supplementary References

1. P. Z. Moghadam, A. Li, S. B. Wiggin, A. Tao, A. G. P. Maloney, P. A. Wood, S. C. Ward and D. Fairen-Jimenez, *Chem. Mater.*, 2017, **29**, 2618-2625.
2. L. Spek, *J. Appl. Cryst.*, 2003, **36**, 7-13.
3. S. A. Moggach, T. D. Bennett, A. K. Cheetham. *Angew. Chem. Int. Edit.,* 2009, 48, 7087-7089.
4. M. J. McGrath, J. N. Ghogomu, N. T. Tsona, J. I. Siepmann, B. Chen, I. Napari and H. Vehkamaki, *J. Chem. Phys.*, 2010, **133**, 084106
5. W. Morris, C. J. Stevens, R. E. Taylor, C. Dybowski, O. M. Yaghi and M. A. Garcia-Garibay, *J. Phys. Chem. C.*, 2012, **116**, 13307-13312.
6. O. Shekhah, R. Swaidan, Y. Belmabkhout, M. du Plessis, T. Jacobs, L. J. Barbour, I. Pinnau and M. Eddaoudi, *Chem. Commun.*, 2014, **50**, 2089-2092.
7. J. L. C. Rowsell, E. C. Spencer, J. Eckert, J. A. K. Howard and O. M. Yaghi, *Science*, 2005, **309**, 1350-1354.
8. L. H. Xie, J. B. Lin, X. M. Liu, Y. Wang, W. X. Zhang, J. P. Zhang and X. M. Chen, *Inorg. Chem.*, 2010, **49**, 1158-1165.
